# Recent Trends in Bioinspired Metal Nanoparticles for Targeting Drug-Resistant Biofilms

**DOI:** 10.3390/ph18071006

**Published:** 2025-07-05

**Authors:** Devaraj Bharathi, Jintae Lee

**Affiliations:** School of Chemical Engineering, Yeungnam University, 280 Daehak-Ro, Gyeongsan 38541, Republic of Korea

**Keywords:** antibiofilm, biofabrication, plants, metal nanoparticles, microbes

## Abstract

Multidrug-resistant (MDR) biofilm infections characterized by densely packed microbial communities encased in protective extracellular matrices pose a formidable challenge to conventional antimicrobial therapies and are a major contributor to chronic, recurrent and device-associated infections. These biofilms significantly reduce antibiotic penetration, facilitate the survival of dormant persister cells and promote horizontal gene transfer, all of which contribute to the emergence and persistence of MDR pathogens. Metal nanoparticles (MNPs) have emerged as promising alternatives due to their potent antibiofilm properties. However, conventional synthesis methods are associated with high costs, complexity, inefficiency and negative environmental impacts. To overcome these limitations there has been a global push toward the development of sustainable and eco-friendly synthesis approaches. Recent advancements have demonstrated the successful use of various plant extracts, microbial cultures, and biomolecules for the green synthesis of MNPs, which offers biocompatibility, scalability, and environmental safety. This review provides a comprehensive overview of recent trends and the latest progress in the green synthesis of MNPs including silver (Ag), gold (Au), platinum (Pt), and selenium (Se), and also explores the mechanistic pathways and characterization techniques. Furthermore, it highlights the antibiofilm applications of these MNPs emphasizing their roles in disrupting biofilms and restoring the efficacy of existing antimicrobial strategies.

## 1. Introduction

**Figure 1:**
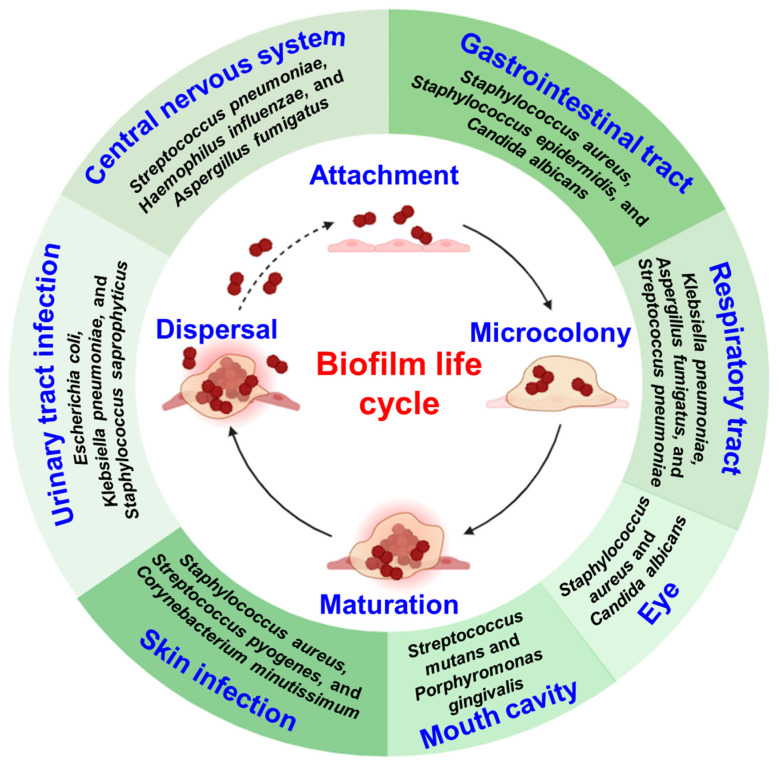
Overview of the biofilm life cycle, highlighting major stages including initial attachment, microcolony formation, maturation and dispersal, along with associated infections. Key microbial species linked to each infection are shown.

Biofilms are structured communities of microorganisms that adhere to surfaces and are embedded in a self-produced matrix of extracellular polymeric substances (EPS) [1,2]. This complex matrix composed of polysaccharides, proteins, nucleic acids and lipids, which offers structural integrity and protective shielding to the embedded microorganisms. The formation of biofilms typically occurs in several stages [3,4,5]: (i) Attachment: Initially, free-floating microorganisms such as bacteria encounter a surface suitable for attachment. These can be biotic or abiotic surfaces including medical implants, industrial pipelines and natural substrates. The initial attachment is facilitated by weak van der Waals forces or electrostatic interactions. (ii) Colonization: Upon attachment microorganisms start to proliferate and form microcolonies on the surface. They produce EPS, which help to anchor the cells to the surface and create a protective environment. (iii) Maturation: As the biofilm continues to develop the microcolonies grow and merge, forming a mature biofilm structure. This structure consists of channels and voids that facilitate nutrient and waste exchange among the microbial community. (iv) Dispersal: Eventually, biofilms undergo dispersal, where cells detach from the biofilm and become free-floating once again. Dispersal can occur actively through the production of enzymes or passively due to environmental disturbances (Figure 1).

While biofilms can have beneficial applications such as wastewater treatment and bioremediation [6], they also present several dangers including the following: (i) Infections: Biofilm-associated infections are a significant concern for human health. Microorganisms within biofilms demonstrate increased resistance to antimicrobial treatments and the host immune system making them challenging to treat [7,8]. (ii) Medical Device Contamination: Biofilm formation on medical devices such as catheters, prosthetic implants and surgical instruments often results in device malfunction, compromised treatment efficacy, and secondary infections. The presence of biofilms on medical surfaces necessitates stringent cleaning and sterilization protocols to prevent contamination [9]. (iii) Industrial Damage: In industrial settings, biofilms can cause biofouling and corrosion of equipment and infrastructure. Biofouling occurs when biofilms accumulate on surfaces such as ship hulls, water pipelines, and heat exchangers, leading to reduced operational efficiency and increased maintenance costs [10]. (iv) Environmental Impact: Biofilms play a crucial role in nutrient cycling and ecosystem dynamics. However, certain types of biofilms such as harmful algal blooms (HABs) can have detrimental effects on aquatic ecosystems, leading to fish death, habitat degradation and economic losses in industries such as aquaculture and tourism [11].

## 2. Nanotechnology Approaches in Combatting MDR Biofilms

Biofilm formation is a pervasive phenomenon in both natural and artificial environments, presenting significant challenges in various fields including medicine, industry and environmental management [9]. Traditional antimicrobial agents often struggle to effectively eradicate biofilms due to their complex structure and increased resistance [12,13]. Metal-based nanoparticles are emerging as potent alternatives to traditional antimicrobial agents particularly in combating MDR biofilms [14]. Among these, MNPs consistently outperform metal oxide nanoparticles in disrupting biofilm structures [15]. This superior efficacy derives from their enhanced metal ion release, which is a key factor in antimicrobial action. For example, AgNPs emit Ag^+^ ions capable of infiltrating the biofilm’s EPS and interfering with vital cellular components such as membranes, proteins, and nucleic acids [16]. Conversely, many metal oxide nanoparticles release ions more slowly or in limited quantities, reducing their immediate impact on biofilms [17].

Additionally, MNPs exhibit higher surface energy and reactivity that enable deeper penetration into biofilm layers and facilitating interactions with both bacterial cells and the EPS [18]. This allows MNPs to target not only surface bacteria but also bacteria which are deeply embedded within the biofilm, which are typically resistant to conventional therapies. MNPs also employ a multifaceted antimicrobial mechanism including the generation of reactive oxygen species (ROS), membrane disruption, protein denaturation and DNA interference [19]. Metal oxide nanoparticles like ZnO and TiO_2_ also produce ROS; however, their activity often depends on specific environmental triggers such as UV exposure or pH changes, limiting their effectiveness under physiological conditions [20]. MNPs maintain their functionality under standard biological environments thus enhancing their clinical applicability. Furthermore, MNPs are highly amenable to surface modification with bioactive agents such as antibiotics, peptides and plant-derived compounds [21,22,23]. This enhances their targeting efficiency and contributes to synergistic antimicrobial outcomes. Although metal oxide nanoparticles can also be functionalized their generally lower surface reactivity may pose challenges for effective conjugation. Based on this significance, the objective of this review is to provide an updated overview of the bio-inspired synthesis of MNPs specifically Ag, Au, Se, Pt NPs, and their current status in combating biofilms formed by drug-resistant bacterial and fungal pathogens. By focusing on these four MNPs for combating drug-resistant biofilms, this review distinguishes itself from earlier studies [14,19,24,25,26,27]. Special emphasis is placed on elucidating their therapeutic mechanisms, addressing challenges in clinical translation and identifying future directions in this evolving field.

## 3. Overview of Metal Nanoparticles Synthesis

MNPs play a critical role in ongoing research within the fields of nanotechnology, materials and biomedical science. Consequently, the synthesis of these nanoparticles has become a subject of significant interest. The unique surface area-to-volume ratio of MNPs and their composite nanomaterials imbues them with exciting physico-chemical, electrical, magnetic, biological and catalytic capabilities. These unique properties are being investigated for diverse applications in health, agriculture and other industries [28,29]. Moving beyond chemical and physical methods, researchers now highlight biofabrication using living organisms or their compounds as an alternative for MNPs synthesis [30]. Despite being effective, wet chemical methods for making MNPs can produce hazardous waste and the chemical agents used in chemistry-based methods such as sodium borohydride and hydrazine hydrate, which may stay adsorbed at the surface of the MNPs, and ultimately limits their applications, particularly in medicine [31]. The cytotoxicity, carcinogenicity and genotoxicity of certain compounds such as hydrazine, are well recognized [32]. Physical methods are energy-intensive and costly with low yields, whereas chemical methods may involve toxic byproducts [33]. Biological processes offer a secure, affordable and environmentally friendly pathway for the synthesis of MNPs that does not include any expensive or high-tech requirements, thus resolving the contradictory needs when balancing energy, cost, toxicity and eco-friendliness [34].

## 4. Bio-Inspired Synthesis of MNPs

**Figure 2:**
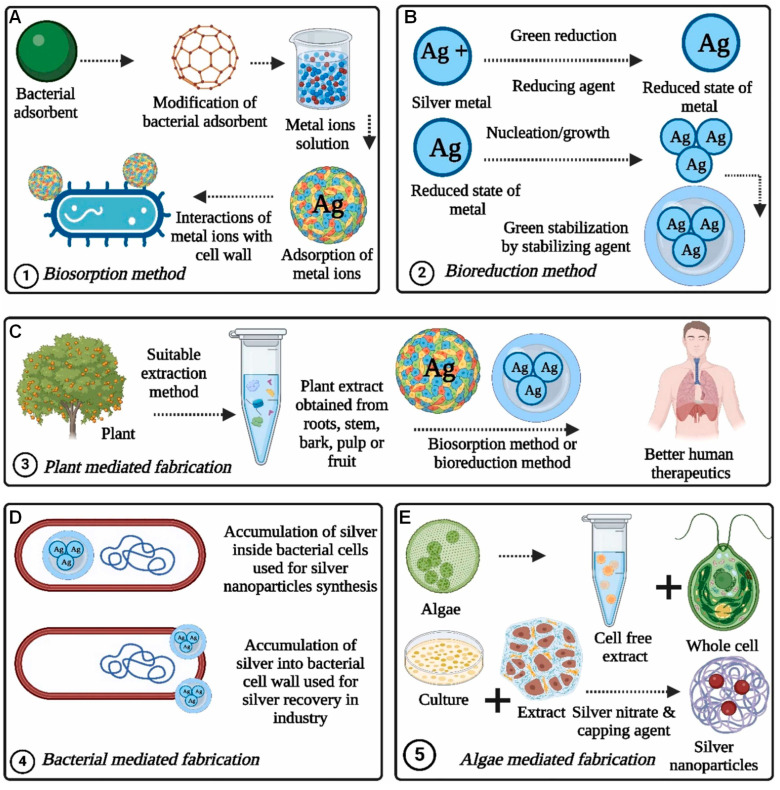
Overview of AgNPs synthesis approaches including the following: (**A**) biosorption-based techniques, (**B**) bioreduction pathways, (**C**) plant-assisted synthesis, (**D**) bacteria-driven methods and (**E**) algae-mediated fabrication. Reprinted with permission from Kumar et al. [45]. Copyright Elsevier.

The bio-inspired synthesis of MNPs is increasingly being recognized as a sustainable alternative to traditional chemical and physical fabrication methods [34]. These biofabrication techniques are favored for their environmental compatibility, lower production costs and higher biological safety [35]. Biosorption is a technique that harnesses the metal-binding capabilities of microbial cell walls, where naturally occurring or surface-engineered functional groups aid in metal ion attachment (Figure 2A) [36]. Bioreduction another well-studied approach, it involves microbial enzymes or metabolic byproducts converting metal ions into their elemental form, while naturally occurring molecules simultaneously or subsequently act as capping agents to stabilize them (Figure 2B) [37]. In the case of plant-based synthesis, solvent extracts derived from various plant parts such as leaves, flowers, fruits and roots serve as both reducing and stabilizing components, making the resulting nanoparticles especially promising for medical applications due to their minimal cytotoxicity (Figure 2C) [34]. Microorganisms, particularly bacteria play a central role in metal accumulation, which can occur either intracellularly or on the cell surface, offering valuable opportunities for nanoparticle synthesis and metal recovery in biotechnological applications (Figure 2D) [38]. Algal species used as intact organisms or via cell-free extracts provide another versatile system for producing nanoparticles both intra- and extracellularly (Figure 2E) [39]. Recent advancements have expanded the pool of biological resources incorporating organisms from extreme environments and also genetically modified organisms (GMOs) that offer the potential to generate nanoparticles with enhanced structural and functional stability [40]. The optimization of environmental and process parameters such as metal salt concentration, pH, incubation time and temperature has led to improved control over nanoparticle attributes including size, morphology and dispersity [41]. An enhanced understanding of the biosynthesis process has been supported by molecular and analytical tools such as omics technologies and spectroscopic analyses, which are helping to elucidate the complex roles of biomolecules in nanoparticle generation [42]. Moreover, the application of genetic and synthetic biology is enabling the engineering of microbes with higher yields and selective synthesis [43]. Concurrently, the biofabrication of multifunctional nanoparticles is opening new avenues in healthcare, diagnostics and environmental protection [44]. The following sections provide a comprehensive overview of individual MNPs synthesis facilitated by plants, microorganisms and biomolecules, highlighting the distinct mechanisms and characteristics associated with each biological system.

## 5. Plant-Mediated Synthesis of MNPs

Over the past decade, plants have emerged as a highly effective and environmentally friendly method for synthesizing MNPs. Plants have demonstrated their suitability for the substantial production of MNPs due to their ability to offer faster, more convenient, cost-effective and reliable synthesis processes [46]. This approach utilizes plant extracts rich in phytochemicals, which act as both reducing and stabilizing agents in the conversion of metal salts into NPs [47]. Recently, Eltaweil et al. [48] demonstrated the biofabrication of PtNPs using *Atriplex halimus* leaf extract. The extract of *A. halimus* effectively facilitated the reduction of platinum ions, resulting in the formation of metallic platinum.

The synthesis method generally entails combining the plant extract with the aqueous solution of the metal salt. The process may exhibit certain variations depending on the specific characteristics of the plant and the MNPs to be synthesized [49]. Overall, the process is environmentally friendly and energy-efficient, and lacks hazardous chemicals, thus demonstrating a sustainable approach [50]. Considerable attention has been directed towards the synthesis of MNPs from plants. However, few studies aim to elucidate the precise mechanism and biochemical pathways involved in this synthesis process. The precise mechanism underlying the synthesis of MNPs using plant extracts has not yet been fully determined [51]. Nevertheless, it is widely acknowledged among researchers that phytochemicals play a crucial role in the development of nanoparticles [52]. Plants possess a diverse range of secondary metabolites including terpenoids, flavonoids, alkaloids, sterols, polyphenols, saponins, polysaccharides, and proteins. These compounds play a crucial role in the bioreduction of metal ions, leading to the formation of MNPs [53]. Recently, Karan et al. [54] illustrated a hypothetic approach to synthesizing AgNPs using *Sambucus ebulus* leaf extract. The leaf extract of *S. ebulus* consists of abundant bifunctional compounds including hesperidin, rutin, isoquercitrin, and *o*-coumaric acid. Hesperidin and rutin acted as reducing and stabilizing agents for the synthesis of AgNPs. In another recent study, Susanna et al. [55] synthesized AuNPs using *Nothapodytes foetida* through ultrasonication with the biofabrication method and demonstrated that the presence of various phytocompounds in *N. foetida* such as flavonoids, alkaloids, phenols, proteins, tannins, and carbohydrates reduced Au^3+^ to Au^0^ NPs. Similarly, Keshtmand et al. [56] developed SeNPs from *Artemisia chamarmelifolia* using an effective and economical green procedure. The leaf extract of *A. chamaemefolia* contains numerous chemical compounds that have the potential to function as both reducing agents and capping agents, and thus facilitate the successful synthesis of stable SeNPs.

The synthesis of MNPs through plant-based methods typically progresses through three key stages [57]. Initially, during the activation stage, metal ions are reduced and initiate the formation of atomic clusters. This is succeeded by the growth stage, where these early nanoclusters undergo aggregation and maturation resulting in larger particles that are energetically stable. The process concludes with the termination stage, in which nanoparticles attain a stable structure and defined morphology influenced by the surrounding biomolecules. Although this general framework is widely accepted, the underlying biochemical pathways and kinetics remain not fully understood. One of the primary challenges arises from the diverse composition of plant extracts, which can vary significantly depending on species, extraction conditions, and environmental influences [58,59]. This complexity makes it difficult to attribute nanoparticle formation to specific phytochemicals. Consequently, researchers are increasingly examining how variables such as pH, temperature, metal salt concentration, and reaction time affect reduction mechanisms and particle development [60,61]. Recent trends in the field highlight the necessity for methodological standardization to ensure reproducibility and scalability. In addition, there is growing interest in employing computational tools and machine learning models to anticipate and modulate nanoparticle features with greater precision [62]. This interdisciplinary focus is paving the way for practical applications especially in the medical field, where plant-derived MNPs show promise in targeted drug delivery for antimicrobial therapies [63]. Several studies have been conducted in recent years to explore the biofabrication of MNPs for various kinds of biomedical applications. These studies have utilized various plant components such as seeds, fruit, root systems, flowers, leaf, and stems as shown in Table 1.

## 6. Microbial Synthesis of MNPs

Microbes possess an extraordinary capability for synthesizing highly specialized MNPs. The microbial synthesis of various MNPs including Ag, Au, Se, and Pt NPs, using bacteria, fungi, yeast, and algae has been reported (Table 2). MNPs fabrication using microbial cells mostly follows either intracellular or extracellular methods. The subsequent section provides comprehensive information about the different microbial methods used for the synthesis of MNPs from microorganisms.

### 6.1. Intracellular Synthesis of MNPs

The microbial accumulation of metals during the processes of bioleaching and bioaccumulation has been known for decades [92]. During the natural biomining process metal ions are transported inside the cell and reduced to the nanoscale range via contact with microbial enzymes and metabolites [93]. Several groups of microorganisms have already been discussed for the intracellular biogenic reduction of metal ions to MNPs [94]. For intracellular synthesis, microbial cells are inoculated in metal ion-containing medium and incubated for the synthesis of respective MNPs under desired microbial growth conditions. During the incubation period, microbial cells absorb metal ions through enzymatic or ion diffusion mechanisms [95]. In this context, microbial enzymes and ions act as reductants for the conversion of metal ions to MNPs and the reduced NPs stored in the cytoplasm, cell wall, and cell membrane of microorganisms. Organisms living in gold mines are better equipped to withstand gold toxicity and generate AuNPs more effectively [96]. When *Acinetobacter* sp. SW30 was incubated with varying concentrations of gold chloride and cell density, the color of AuNP-containing colloidal solution varied dramatically, indicating the formation of AuNPs with diverse sizes and shapes. During synthesis, amino acids are involved in the reduction in gold salts, while amide groups aid in the stability of AuNP [97]. In addition, nanocrystals of Ag, Au and their alloys have been biosynthesized inside the lactic acid bacteria cells [98].

The intracellular synthesis of MNPs using microorganisms such as *Pseudoalteromonas* sp., *Gluconacetobacter liquefaciens*, *Saccharomyces cerevisiae*, *Enterococcus faecalis*, *Acinetobacter calcoaceticus*, and many more have been reported [99,100,101,102,103]. The literature reports have stated that cofactors like nicotinamide adenine dinucleotide hydrogen (NADH), NADH-dependent enzymes, and nitrate reductase enzymes play a vital role in the intracellular synthesis of MNPs [29]. However, the separation and recovery of reduced MNPs from inside a microorganism has limited the intracellular synthesis methods.

### 6.2. Extracellular Synthesis of MNPs

The extracellular synthesis of MNPs has received greater attention than intracellular synthesis due to its reduced downstream processing steps, easy purification, and high product yield [104]. Extracellular synthesis occurs outside the cells via different process such as (1) being synthesized intracellularly and later transported out of the microbial cell, (2) being synthesized on the surface of the cell wall, and (3) using a cell supernatant of microbial cultures [92,105,106]. The synthesis of MNPs using cell-free supernatant is very common and has gained worldwide popularity. Several microbial metabolites such as carbohydrates, proteins, and extracellular enzymes have acted as reducing agents for the biogenic reduction in MNPs [103,107]. Recently, Prema et al. [108] showed that the extracellular metabolites of *Lactobacillus plantarum* were used to synthesize AgNPs for antibacterial and antioxidant activities.

Several researchers have used microbial biomass for the green synthesis of MNPs. For example, Wilson et al. [109] investigated the green synthesis of AgNPs using the extracellular biomass of *Bacillus subtilis* and found that the presence of extracellular metabolites in biomass acted as reductant for the conversion of AgNO_3_ to AgNPs. *Trichoderma harzianum* was used as a reductant for the synthesis of SeNPs [110]. The results revealed that extracellular metabolites such as carbohydrates, sugars, organic acids, and amino acids produced by the fungi caused the bioreduction of sodium selenite to SeNPs [110]. However, the molecular mechanism of the microbial supernatant-assisted biosynthesis of MNPs is unknown. Recent advancements in microbial-mediated nanoparticle synthesis have increasingly focused on fine-tuning synthesis parameters including metal ion concentration, pH, temperature, and incubation duration to achieve precise control over nanoparticle size, shape, and distribution [111]. At the same time, the application of advanced biochemical and molecular techniques has deepened our understanding of microbial metabolic pathways shedding light on the specific roles played by different microbial species in nanoparticle formation [38]. A growing area of interest lies in the use of extremophilic microorganisms sourced from harsh and unique environments, which often possess distinctive biochemical capabilities and produce nanoparticles with enhanced stability and novel features [112]. Furthermore, synthetic biology and genetic engineering are set to significantly impact this field by enabling the development of custom-engineered microbial strains with improved efficiency and selectivity for nanoparticle synthesis. These engineered microbes can be designed to biosynthesize nanoparticles with precise structural and functional attributes, which are tailored to specific applications [113]. To bridge the gap between laboratory research and industrial application, significant efforts are also being directed toward bioreactor design and process engineering, ensuring that microbial synthesis can be scaled up in sterile and controlled environments with consistent yield and quality.

**Table 2:** Microorganism-mediated synthesis of MNPs: type, shape, and size characteristics.

Microorganisms	Description	Extracellular/ Intracellular	Types of NPs	Shape	Size (nm)	References
*Lysinibacillus* sp.	Bacteria	Extracellular	Ag	Quasi-spherical	7.5–14.7	Pernas-Pleite et al. [114]
*Cupriavidus* sp.	Bacteria	Extracellular	Ag	Spherical	10–50	Ameen et al. [115]
*Phormidesmis communis* Strain AB_11_10	Bacteria	Extracellular	Au	Quasi-spherical, triangular, and rectangular	2–28	Hamida et al. [116]
*Streptomyces olivaceus* (MSU3)	Bacteria	Extracellular	Ag	Spherical	12.3	Sanjivkumar et al. [117]
*Alcaligenes* sp.	Bacteria	Extracellular	Ag	Spherical	30–50	Divya et al. [118]
*Penicillium chrysogenum* MF318506	Fungi	Extracellular	Ag	Spherical	9.75–25.21	Abd El Aty et al. [119]
*Desmodesmus* sp.	Algae	Intracellular	Ag	Spherical	15–30	Dağlıoğlu and Öztürk [120]
*Portieria hornemannii*	Algae	Extracellular	Ag	Spherical	60–70	Fatima et al. [121]
*Micrococcus yunnanensis* J2	Bacteria	Extracellular	Au	Spherical	53.8	Jafari et al. [122]
*Ganoderma applanatum*	Fungi	Extracellular	Au	Cubic	18.7	Abdul-Hadi et al. [123]
*Pseudoalteromonas* sp. Bac178	Bacteria	Intracellular	Au	Spherical	26.12	Patil et al. [100]
*Gluconacetobacter liquefaciens*	Bacteria	Intracellular	Au	Spherical	11,232	Liu et al. [99]
*Paracoccus haeundaensis* BC74171*^T^*	Bacteria	Extracellular	Au	Spherical	20.93 ± 3.46	Patil et al. [124]
*Sargassum plagiophyllum*	Algae	Extracellular	Au	Spherical	65.87	Dhas et al. [125]
*Parmelia sulcata*	Fungi	Extracellular	Au	Spherical	54	Gandhi et al. [126]
*Aspergillus flavus*	Fungi	Extracellular	Au	Spherical	12	Abu-Tahon et al. [127]
*Streptomyces griseoruber*	Bacteria	Extracellular	Se	Spherical	100–250	Ranjitha et al. [107]
*Saccharomyces cerevisiae*	Yeast	Intracellular	Se	Spherical	50	Faramarzi et al. [101]
*Enterococcus faecalis*	Bacteria	Intracellular	Se	Spherical	29–195	Shoeibi and Mashreghi [102]
*Mariannaea* sp. HJ.	Fungi	Intra and Extracellular	Se	Spherical	45.19 and 212.65	Zhang et al. [128]
*Fusarium oxysporum*	Fungi	Extracellular	Pt	Cubical, spherical, and triangular	25	Gupta et al. [129]
*Pseudomonas kunmingensis* ADR19	Bacteria	Extracellular	Pt	Spherical	3.95	Eramabadi et al. [130]
*Psychrobacter faecalis* FZC6	Bacteria	Extracellular	Pt	Spherical	2.49	
*Vibrio fischeri* NRRL B-11177	Bacteria	Extracellular	Pt	Spherical	3.84	
*Jeotgalicoccus coquinae* ZC15	Bacteria	Extracellular	Pt	Spherical	5.74	

## 7. Biomolecule-Mediated Synthesis of MNPs

The biomolecule-mediated synthesis of MNPs represents a promising green alternative to conventional methods, it utilizing naturally occurring compounds such as proteins, enzymes, amino acids, polysaccharides, flavonoids, phenolics, and vitamins to facilitate nanoparticle formation [131]. These biomolecules act as both reducing agents, by converting metal ions into their elemental states, and stabilizing agents, preventing aggregation through interactions with functional groups such as hydroxyl, carboxyl, amine, and thiol groups [45,132]. This dual functionality allows for the controlled synthesis of MNPs with tailored size, shape, and surface characteristics. For instance, proteins and enzymes contribute redox-active amino acids for reduction and provide a stabilizing matrix, while polysaccharides and organic acids assist in forming biocompatible, water-dispersible particles [133]. For example, Theodosiou et al. [134] demonstrated the successful synthesis of AuNPs using 17 different L-amino acids as reducing agents. Aromatic amino acids acted as rapid reductants, effectively facilitating the formation of AuNPs. In contrast, hydroxylic and basic amino acids exhibit significantly slower reduction kinetics during AuNPs synthesis. Most aliphatic amino acids generated AuNPs with comparable features, although Isoleucine uniquely produced hollow nanospheres. Notably, AuNPs synthesized using aspartic acid and asparagine displayed similar physicochemical characteristics to those obtained via citrate-mediated synthesis, which is widely regarded as the benchmark method for producing plasmonic AuNPs. Another study by Ponjavic et al. [135] showed the successful green synthesis of PtNPs using bacterial nanocellulose as a reducing and capping agent. Plant-derived biomolecules not only reduce metal ions effectively but also impart bioactive properties to the nanoparticles, making them suitable for antimicrobial and antioxidant applications. Recently, quercetin extracted from *Arctium lappa* was utilized for the synthesis of AgNPs and AuNPs. In this process, laser ablation was employed to generate nanoparticles, while the extracted quercetin functioned as a natural reducing and stabilizing agent [136].

Biomolecule-based synthesis is particularly advantageous due to its high biocompatibility, eco-friendliness, and potential for fine control over nanoparticle morphology, making it well suited for biomedical, environmental, and agricultural applications [30]. The mild synthesis conditions often involve ambient temperature and pressure, further supporting energy conservation and operational safety. However, challenges remain in achieving consistent results, understanding the precise mechanisms involved, and scaling up production, especially when using complex natural mixtures. Table 3 presents an overview of biomolecule-mediated MNPs synthesis with corresponding types, shapes, and sizes.

## 8. MNP Characterization Methods

After the biosynthesis of MNPs, a critical next step involves the identification and characterization of various parameters including size, morphology, chemical composition, surface area, and crystallography. The key characterization techniques employed for MNPs include UV–visible spectrophotometry, fourier-transform infrared spectroscopy (FTIR), scanning electron microscopy (SEM), transmission electron microscopy (TEM), energy dispersive spectroscopy (EDS), X-ray diffraction (XRD), zeta potential and dynamic light scattering (DLS) analysis.

### 8.1. Visual Inspection and UV–Visible Spectrophotometry

The formation of MNPs can be initially identified by observing a color change in the bio-medium–metal–salt mixture. The specific color transformation depends on the type of MNPs being synthesized. In the case of AgNPs, a brown hue develops, as seen when using *Coffea arabica* extract [150]. AuNPs appear purple when derived from the carthamidin compound [151]. PtNPs synthesized via *Nymphaea tetragona* have a dark brown or black coloration [152] while SeNPs obtained from *Moringa oleifera* exhibit a distinct green color [153].

UV–visible spectroscopy is commonly used to confirm the formation of MNPs by detecting surface plasmon resonance (SPR) in the absorbance spectra [26]. This technique demonstrates the reduction of metal ions into their corresponding nanoparticles such as Ag^+^ to Ag^0^ for AgNPs, Au^3+^ to Au^0^ for AuNPs, SeO_3_^2−^ to Se^0^, and Pt^2+^ to Pt^0^ for PtNPs facilitated by biomolecules from plant extracts or microbial cells and their products. The characteristic SPR peaks appear at specific wavelengths: AgNPs typically absorb between 400 and 450 nm [68,76], AuNPs between 510 and 570 nm [154], and PtNPs and SeNPs between 250 and 300 nm, respectively [155,156,157].

### 8.2. FTIR Analysis

The main purpose of FTIR analysis is to identify the functional groups attached to the surface of MNPs. Additionally, it is beneficial for examining the surface chemistry of the produced nanoparticles. The absorption bands detected at 3300–3400 cm^−1^, 2940 cm^−1^, 1600–1700 cm^−1^, 1350–1450 cm^−1^, and 1000–1100 cm^−1^ correspond to different functional groups. These include O-H and C-H vibrations in aromatic compounds, C=O stretching in carbonyl groups, and bonds such as C-C, C-O-C, C-N, and C-O [158,159]. Additionally, the N-H vibration of amides and C=C bonds contribute to these spectral features [160]. These functional groups are commonly associated with phytochemicals like polyphenols, proteins, alcohols, terpenoids, enzymes, and alkaloids. When comparing FTIR spectra of nanoparticles with plant extracts or secondary metabolites a noticeable shifts or reductions in peak intensities are observed, thus indicating the role of phytochemicals in both the formation and stabilization of MNPs [161].

### 8.3. SEM, TEM, and EDX Analysis

SEM and TEM analyses provide valuable insights into the morphological and surface characteristics of MNPs. For example, AgNPs obtained from *Salvia sclarea* flower extract showed spherical nanoparticles with a particle size in the 30–40 nm range [162]. On the other hand, AuNPs synthesized from gum arabic with cinnamon extract exhibited spherical, diamond, and hexagonal shapes with particle sizes ranging from 12 to 17 nm [163]. Another example is SeNPs synthesized from *Hericium erinaceus* polysaccharide with TEM analysis demonstrating the formation of uniformly sized particles of about 11 nm that were spherical in shape [157]. SEM and TEM can also reveal the coating of biosynthesized NPs, which is generally composed of plant phytochemicals that cover the particles’ surface [164].

Further, the elemental composition can be determined using EDX analysis. Strong absorption peaks at 3 keV, 2.12 keV, 2.05 keV, and 1.37 keV indicate the binding energies of Ag, Pt, Au, and Se, respectively [41,48,97,165]. In the case of biosynthesized NPs, additional elements that may be present such as carbon, oxygen, and nitrogen, which may resulted from plant phytochemicals or biomolecules that act as capping elements on the surface of MNPs [26].

### 8.4. XRD Analysis

X-ray diffraction (XRD) is a widely used technique to assess the crystalline nature and structural characteristics of nanoparticles. Based on Bragg’s law of diffraction and standard reference data from the Joint Committee on Powder Diffraction Standards (JCPDS), the presence of sharp and well-defined peaks in the XRD spectrum confirms the crystallinity of nanoparticles. For AgNPs, prominent peaks are observed at 2θ values of around 38°, 44°, and 64°, which correspond to the (111), (200), and (220) crystal planes, respectively [162,166]. Mostly, these reflections are indicative of a face-centered cubic (FCC) structure, in agreement with JCPDS No. 65-2871. In the case of AuNPs, characteristic diffraction peaks appear at approximately 38°, 44°, 64°, and 77°, corresponding to the (111), (200), (220), and (311) planes of an FCC lattice, consistent with JCPDS No. 01-089-3697 [151]. For PtNPs, the XRD pattern typically exhibits peaks at 2θ values near 39.7°, 46.2°, 67.4°, and 81.2°, which can be indexed to the (111), (200), (220), and (311) planes of a FCC structure, in line with JCPDS No. 04-0802, confirming the crystalline and metallic nature of PtNPs [48]. SeNPs generally display a different diffraction pattern due to their trigonal crystal structure. XRD peaks are often detected at 2θ values of around 23.5°, 29.7°, 43.5°, and 51.6°, which correspond to the (100), (101), (111), and (201) planes, as per JCPDS No. 06-0362, indicating the presence of well-formed crystalline SeNPs [167].

In addition to revealing structural characteristics, XRD analysis plays a crucial role in assessing the phase purity of nanoparticles. It can detect impurities or the formation of secondary phases, as well as tracking variations in crystallinity that may arise from different synthesis approaches or surface modifications such as capping agents, doping, or thermal treatments. The appearance of unexpected or unassigned peaks in the diffraction pattern often signifies the presence of residual precursors, by-products, or undesired crystalline phases [26,168].

### 8.5. Zeta Potential and Dynamic Light Scattering Analysis

The surface charge of MNPs plays a crucial role in determining their interactions with target sites. To evaluate this surface charge and the electrostatic stability of dispersions in a liquid medium, zeta potential measurements were conducted using a zeta potential analyzer [169]. Zeta potential arises from ionizable or adsorbed groups on the particle surface, providing insight into the surface charge characteristics [169]. This parameter is essential for assessing the aggregation behavior and colloidal stability of MNPs in suspension. A zeta potential value greater than +30 mV or less than −30 mV typically indicates a highly stable dispersion of nanoparticles due to the strong electrostatic repulsion [170]. Conversely, values near 0 mV suggest a higher tendency toward aggregation, as the repulsive forces are insufficient to prevent particle collision and flocculation [171]. In general, zeta potential values tend to be negative and thus reflecting the presence of electrostatic repulsion that helps maintain nanoparticle dispersion stability.

The polydispersity index (PDI) describes the breadth of the particle size distribution. This ranges from 0 to 1, where values close to 0 denote a monodisperse system with uniform particle sizes and values approaching 1 reflect a polydisperse system with a wide size distribution. To measure the hydrodynamic diameter of nanoparticles in suspension, dynamic light scattering (DLS) is employed. This technique detects fluctuations in scattered light intensity caused by the Brownian motion of particles [172]. Notably, variations in nanoparticle size can arise due to surface coatings with biomolecules such as plant extract constituents, which differ in molecular weight and influence the measured hydrodynamic size [173].

## 9. Antibiofilm Properties of MNPs

MNPs have attracted attention as powerful agents in the disruption of biofilm-related infections largely due to their distinctive physicochemical features. Among them, Ag, Au, Se, and Pt exhibit marked antibiofilm activity via multiple modes of action. AgNPs are especially noted for releasing Ag^+^ ions that can infiltrate the biofilm matrix, compromise membrane structure, induce oxidative stress, and hinder communication systems like quorum sensing that are vital for biofilm development [133]. AuNPs exhibit excellent biocompatibility and can be effectively functionalized with drugs or bioactive ligands to enhance their antibiofilm efficacy. Their photothermal properties further enable the physical disruption of biofilm structures upon laser irradiation, making them a versatile and promising tool in antimicrobial strategies [174]. SeNPs operate by disturbing redox homeostasis in microbes, generating ROS, and reducing microbial adhesion, all while maintaining a relatively low toxicity, making them suitable for drug-resistant antibiofilm therapy [175]. PtNPs, owing to their strong catalytic properties and nanozyme-like behavior and continuously produce ROS, which disrupt microbial membranes and enhance the efficacy of antibiotics [176]. These MNPs collectively offer innovative strategies for preventing and dismantling resilient biofilms, especially in the context of drug-resistant microbial strains and surface-associated infections. The following section discusses the antibiofilm activity of individual MNPs.

### 9.1. Antibiofilm Efficacy of Biosynthesized AgNPs

Among the various MNPs explored for antibiofilm therapy, AgNPs have emerged as one of the most promising due to their nanoscale properties and multifaceted mechanisms of microbial inhibition. AgNPs exhibit potent antibiofilm activity primarily through the release of Ag^+^, which interacts with microbial membranes, disrupts EPS, and induces oxidative damage [158]. These ions can easily diffuse through the extracellular matrix of biofilms and interact with sulfur- and phosphorus-containing biomolecules within microbial cells [24]. The development of ROS by AgNPs leads to lipid peroxidation, protein denaturation, and DNA fragmentation, ultimately causing microbial cell death [177]. Moreover, their nanoscale size allows them to approach and interact with biofilm surfaces more efficiently than larger particles, enhancing their ability to penetrate and disrupt mature biofilms [178]. Beyond these effects, AgNPs are capable of interfering with the initial stages of biofilm formation. They alter the physicochemical characteristics of surfaces such as charge and hydrophobicity, thereby reducing bacterial attachment. AgNPs also target quorum sensing, the bacterial signaling mechanism that regulates gene expression involved in biofilm maturation and virulence. By disrupting these pathways, AgNPs prevent biofilm progression and reduce microbial coordination within the colony [179].

**Figure 3:**
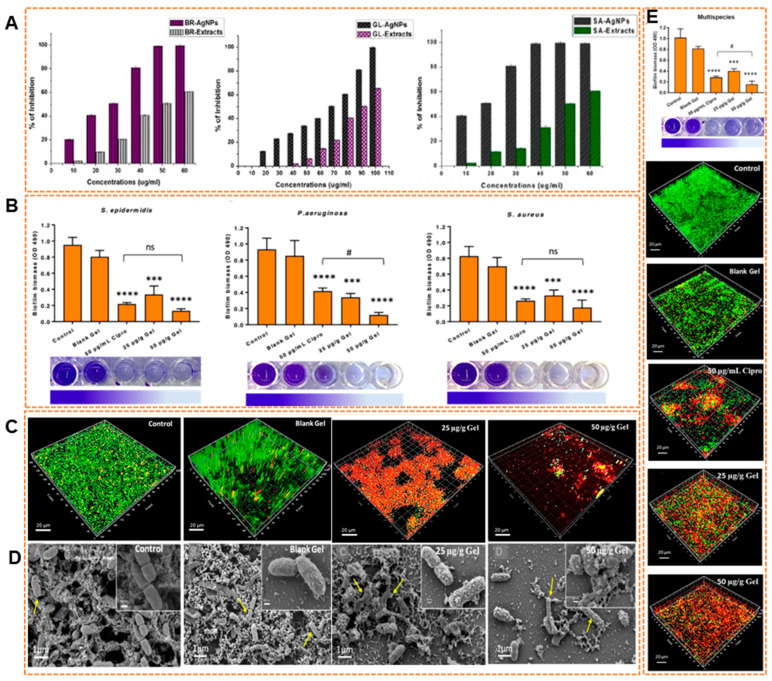

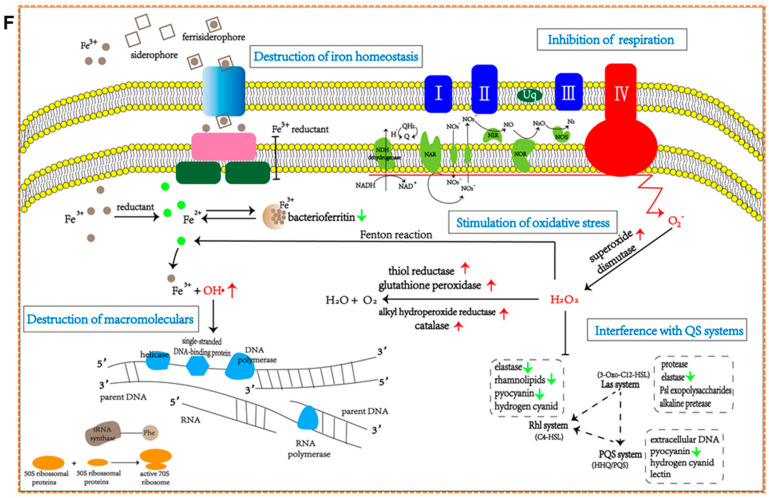
(**A**) Antibiofilm activity of biosynthesized AgNPs against *P. aeruginosa*. Reprinted with copyrights, Mohanta et al. [179]. (**B**) Eradication of *S. epidermidis*, *P. aeruginosa*, and *S. aureus* biofilms, along with representative images illustrating the intensity of CV staining. (**C**,**D**) CLSM and SEM images of *P. aeruginosa* biofilm structure before and after treatment with AgNPs gel. (**E**) Eradication of multi-species biofilms with CV staining and their CLSM images before and after treatment. (**F**) Mechanism of action (Red arrows indicate upregulation, whereas green arrows represent downregulation). “ns” indicates a non-significant difference; “#” denotes a significant difference compared to ciprofloxacin (*p* < 0.05). Asterisks indicate statistical significance (*** *p* < 0.001 and **** *p* < 0.0001). Reproduced with permission from Haidari et al. [184] and Zhang et al. [190]. Copyright, American Chemical Society.

The applications of AgNPs in medical fields are rapidly increasing. The coating of biomaterials such as catheters, orthopedic implants, and wound dressings with AgNPs has shown to significantly reduce microbial colonization and biofilm formation [133,180]. In therapeutic settings, AgNPs are being evaluated for their potential in managing chronic infections caused by biofilm-forming pathogens like *Pseudomonas aeruginosa*, *Staphylococcus aureus*, *Escherichia coli*, and *Candida* sp. [181,182]. Importantly, AgNPs have demonstrated the ability to reach and destroy sessile cells within mature biofilms, where many antibiotics fail due to restricted penetration and resistance development [183]. The effectiveness of AgNPs can be enhanced by tuning their synthesis process and surface functionalization. The AgNPs were biosynthesized using *Semecarpus anacardium*, *Glochidion lanceolarium*, and *Bridelia retusa* extracts exhibited strong antibiofilm activity against *P. aeruginosa*, *E. coli*, and *S. aureus* [179]. Among them, *S. anacardium*-derived AgNPs showed the most potent effect with the lowest IC_50_ values. The treatment of *P. aeruginosa* with 100 μg/mL of AgNPs synthesized from *G. lanceolarium* leaf extract for 24 h resulted in a >99% reduction in biofilm formation (Figure 3A). Recently, Haidari et al. [184] demonstrated that the incorporation of ultrasmall AgNPs into a biocompatible Pluronic F-127 hydrogel resulted in highly potent antibiofilm activity. The AgNPs-loaded hydrogel effectively disrupted and eradicated single-species biofilms of *P. aeruginosa*, *S. aureus*, and *S. epidermidis* in a concentration-dependent manner, outperforming ciprofloxacin at equivalent concentrations (Figure 3B). Notably, these antibiofilm effects were achieved at relatively low AgNPs concentrations (25–50 μg/mL) underscoring the potential of this Ag-loaded hydrogel platform as an effective and biocompatible strategy for managing biofilm-associated infections. In addition, confocal laser scanning microscopy (CLSM) and SEM analyses confirmed extensive *P. aeruginosa* biofilm disruption and bacterial cell damage following treatment (Figure 3C). Furthermore, the Ag-hydrogel exhibited significant efficacy against multispecies biofilms, leading to substantial reductions in both biofilm biomass and bacterial viability, and also CLSM analysis confirmed this biofilm reduction (Figure 3D). Figure 3E presents a comprehensive mechanistic model illustrating the antibiofilm effects of AgNPs against multidrug-resistant *P. aeruginosa* biofilms. The figure summarizes that AgNPs target multiple physiological processes, which are critical for biofilm development and maintenance. Specifically, AgNPs inhibit bacterial adhesion and motility, thereby preventing initial biofilm establishment. They induce a strong oxidative stress response by producing ROS, leading to oxidative damage to bacterial cells within the biofilm. Additionally, AgNPs enhance ROS production by releasing silver ions that interact with cellular thiol groups, thus impairing antioxidant defenses and disrupting mitochondrial function. This redox imbalance promotes continuous ROS induction, ultimately amplifying oxidative stress and contributing to their potent antimicrobial activity [185,186]. AgNPs treatment also impairs aerobic and anaerobic respiratory pathways by downregulating key enzymes involved in electron transport and nitrate reduction, weakening the biofilm’s metabolic resilience under hypoxic conditions. Finally, AgNPs interfere with QS systems, leading to the suppression of virulence factor production and further destabilizing the biofilm community [184].

Moreover, functionalizing AgNPs with biocompatible polysaccharides such as polyethylene glycol enhances their dispersion and colloidal stability and facilitates their targeted delivery to infection sites. Such modifications also allow for controlled ion release, reduced cytotoxicity, and enhanced therapeutic efficiency particularly when integrated with existing antimicrobial agents [187]. Velmathi et al. [188] reported the biosynthesis, structural characterization, and antibiofilm potential of AgNPs synthesized using *Deinococcus radiodurans*. In their approach, silver ions were reduced by the bacterial cell free supernatant, yielding stable and spherical AgNPs. These nanoparticles were subsequently modified with PEG and curcumin to create a multifunctional nanocomposite (AgNPs-PEG-Cur). PEG–curcumin conjugation increased hydrodynamic size, indicating effective surface modification. In vitro biofilm inhibition assays demonstrated that the nanocomposite exhibited superior efficacy against biofilms formed by *E. coli* and *S. aureus*, surpassing the effects of either AgNPs or curcumin alone. Notably, biofilm suppression reached as high as 93.6% in *S. aureus* with fluorescent microscopy confirming a significant reduction in viable biofilm-embedded cells. One of the notable advantages of AgNPs is their ability to act synergistically with antibiotics. In such combinations, AgNPs can compromise the biofilm structure and increase the permeability of microbial membranes, allowing for an increase in the intracellular accumulation of antibiotics and restoring their efficacy against resistant strains. For example, Aboelmaati et al. [189] developed biocompatible AgNPs synthesized using *Hibiscus sabdariffa* stem extract and further functionalized them with polydopamine (PDA) to create a versatile platform for antibiotic delivery. These PDA-coated AgNPs were conjugated with two fluoroquinolone antibiotics, moxifloxacin and gatifloxacin, using carbodiimide chemistry to enhance their biofilm-targeting capacity. Their study assessed the conjugates performance against biofilms formed by MDR pathogens including *K. pneumoniae*, *P. aeruginosa*, and *Acinetobacter baumannii*. While unmodified green AgNPs alone (≤50 µg/mL) showed moderate antibiofilm activity, conjugation with antibiotics significantly enhanced their performance. Minimum biofilm inhibitory concentrations (MBICs) for the conjugates were reduced by several fold compared to free antibiotics: up to 75.8-fold for moxifloxacin and 9.4-fold for gatifloxacin against *P. aeruginosa* and *K. pneumoniae*, respectively. Similarly, the minimum biofilm bactericidal concentrations (MBBCs) were lowered considerably, with conjugated systems showing 4.7–37.6-fold increases in bactericidal activity. The enhanced efficacy was attributed to the synergistic effects of the released Ag^+^ ions, ROS production from the PDA-AgNPs complex, and the improved cellular uptake and biofilm penetration facilitated by the nanoscale size of the conjugates. This combined approach not only improves efficacy but also reduces the likelihood of the development of resistance, making AgNPs antibacterial conjugates a promising strategy against drug-resistant biofilms.

### 9.2. Antibiofilm Efficacy of Biosynthesized AuNPs

**Figure 4:**
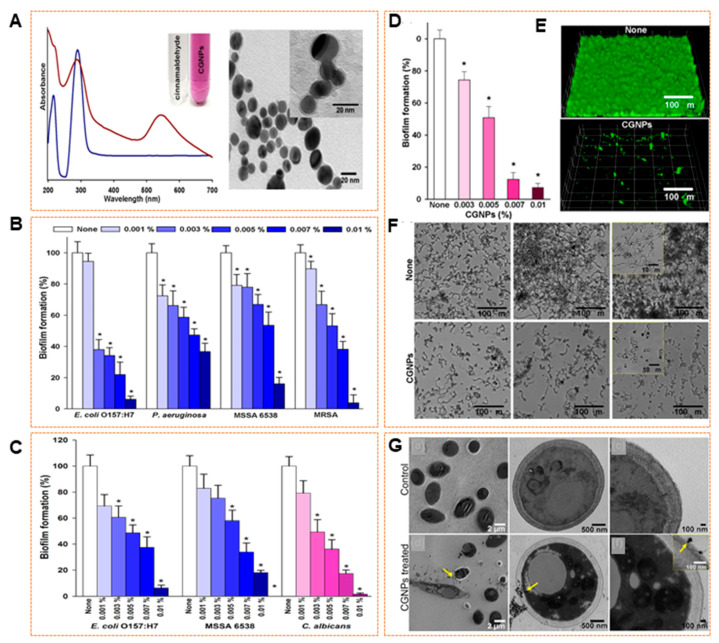
(**A**) UV–visible absorbance spectra (blue line represents cinnamaldehyde, and red line represent CGNPs) and TEM image of CGNPS. (**B**) Antibiofilm activity of AuNPs against *E. coli*, *P. aeruginosa*, MSSA 6538, and MRSA. (**C**) Antibiofilm activity of cinnamaldehyde-AgNPs against *E. coli*, MSSA, and *C. albicans*. (**D**) The effect of AuNPs on biofilm formation of *C. albicans* and their (**E**) CLSM, (**F**) optical microscope, and (**G**) TEM images. Asterisks indicate statistical significance (* *p*< 0.05 versus control). Reproduced with permission from Ramasamy et al. [193]. Copyright, Elsevier.

AuNPs are increasingly recognized in biomedical fields as multifunctional tools, extending beyond their established roles in diagnostics and drug delivery to applications in microbial control. AuNPs demonstrate significant antibiofilm activity driven by their versatile surface chemistry, stable dispersion in the colloidal systems, and outstanding biocompatibility, making them a promising alternative to more cytotoxic agents. [191]. A primary strategy for leveraging AuNPs for biofilm disruption involves their ability to be tailored with diverse therapeutic moieties. Functionalization with antibiotics, peptides, or enzymes enables these particles to act as precision-targeted carriers, facilitating the accumulation of antimicrobial agents at biofilm sites. This localized delivery improves penetration through the EPS and supports sustained therapeutic release within the biofilm microenvironment [192]. Ramasamy et al. [193] reported that AuNPs functionalized with cinnamaldehyde (CGNPs), synthesized through a direct one-pot method, exhibited strong antibiofilm activity. Characterization analysis showed that the AuNPs were spherical and uniformly distributed with an average particle size in the nanoscale range (Figure 4A). Furthermore, at a low cinnamaldehyde concentration (0.01%) the AuNPs effectively inhibited biofilm formation by more than 90% in *E. coli* O157:H7 and MRSA, and 65% and 85% for *P. aeruginosa* and MSSA 6538, respectively (Figure 4B). Additionally, CGNPs also showed a remarkable biofilm inhibition against *E. coli* O157:H7, MSSA 6538, and *C. albicans* (Figure 4C). Furthermore, AuNPs synthesized using 0.01% cinnamaldehyde exhibited biofilm inhibition *C. albicans* (Figure 4D). Confocal and optical microscopy confirmed the structural disruption of biofilms, while TEM imaging revealed the internalization of NPs and intracellular damage (Figure 4E–G). The AuNPs disrupted fungal biofilms by inhibiting hyphal formation in *C. albicans* indicating enhanced penetration and antimicrobial delivery compared to free cinnamaldehyde or unmodified AuNPs. Similarly, Vijayakumar et al. [194] discussed the antibiofilm capabilities of AuNPs synthesized using the marine polysaccharide laminarin against *Aeromonas hydrophila*. The AuNPs demonstrated a concentration-dependent reduction in biofilm biomass with significant inhibition observed at 100 µg/mL. Microscopic evaluation following CV staining revealed a disrupted biofilm architecture compared to untreated controls. Further analyses showed that treatment with AuNPs lowered the bacterial cell surface’s hydrophobicity by 58%. Additionally, AuNPs significantly suppressed the production of EPS, reducing EPS levels by approximately 56% compared to untreated cells.

Moreover, AuNPs have been shown to interfere with bacterial communication systems particularly quorum sensing routes such as lasE and rhlR, which are critical in biofilm maturation and structural coordination [195,196]. Inhibiting these signaling pathways leads to the suppression of EPS production and limits cellular attachment, thereby attenuating biofilm integrity and growth. The AuNPs not only disrupted early biofilm formation but also interfered with filamentation, thereby weakening the structural and invasive capacity of *C. albicans* [197]. A unique advantage of AuNPs is their photothermal responsiveness. When exposed to near-infrared light, they can generate localized heat, disrupting biofilm matrices and compromising microbial cell membranes. This effect is particularly potent when used in conjunction with antibiotics, as the thermal energy enhances permeability and deepens drug access to otherwise shielded biofilm layers [198,199].

AuNPs are also characterized by their chemical inertness and low oxidative reactivity, making them suitable for integration into systems where minimal cytotoxicity is required such as ophthalmic applications, dental materials, or chronic wound therapies. Jawad et al. [200] evaluated the antibiofilm potential of amikacin-conjugated AuNPs for contact lens-associated microbial infections. Using *P. aeruginosa* and *S. pneumoniae* as model pathogens, they found that the Am@GNPs significantly reduced biofilm formation on contact lens surfaces. AFM analysis revealed a marked decrease in biofilm thickness compared to treatments with either free amikacin or unmodified AuNPs. The Am@GNP-treated lenses exhibited average biofilm thickness values of 221 nm for *P. aeruginosa* and 97.5 nm for *S. pneumoniae*, indicating superior biofilm disruption. This enhanced effect was attributed to the synergistic combination of AuNP-mediated surface interactions and the antimicrobial action of amikacin, which together compromised biofilm integrity and cell adhesion. Additionally, AuNPs have been embedded into biocompatible matrices, including hydrogels, polymeric films, and surface coatings effectively reducing microbial colonization on biomedical devices. At the cellular level, they can induce metabolic imbalance and membrane stress in microorganisms, diminishing biofilm biomass without triggering typical resistance mechanisms. These combined actions make AuNPs a promising strategy against drug-resistant biofilm infections.

### 9.3. Antibiofilm Efficacy of Biosynthesized SeNPs

**Figure 5:**
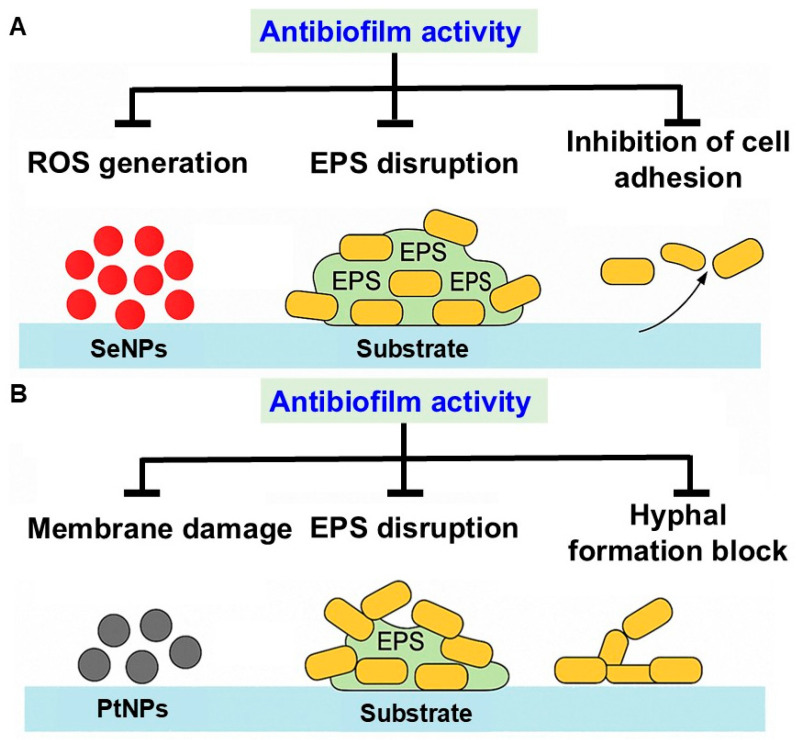
Antibiofilm mechanisms of (**A**) SeNPs and (**B**) PtNPs including ROS generation, metal ion release, membrane disruption, EPS degradation, and gene regulation.

SeNPs have attracted considerable interest due to their effectiveness in combating infections associated with MDR biofilm. SeNPs offer several distinct advantages in targeting drug-resistant biofilms compared to conventional strategies [201]. Their redox-modulating capabilities enable the selective disruption of microbial oxidative stress responses, leading to controlled ROS release that weakens biofilm integrity without harming surrounding tissues [202]. SeNPs also interfere with quorum sensing and inhibit the production of EPS, thereby suppressing biofilm formation and maintenance (Figure 5A).

The antibiofilm efficacy of SeNPs against key pathogens including *P. aeruginosa*, *S. mutans*, *E. coli*, and *C. albicans* has consistently been reported [201,203,204]. SeNPs have been shown to downregulate the expression of QS-related genes such as N-(3-oxododecanoyl)-L-homoserine lactone synthase (LasI), N-butyryl-L-homoserine lactone synthase (RhlI), N-(3-oxododecanoyl)-L-homoserine lactone-responsive transcriptional regulator (LasR), N-butyryl-L-homoserine lactone-responsive transcriptional regulator (RhlR), and multiple virulence factor regulator, which are essential for microbial communication, virulence, and biofilm maturation [205]. For example, SeNPs synthesized using a cell-free supernatant of *Streptomyces* isolate S91 showed potential antibiofilm activity against *P. aeruginosa* (PA01). It exhibited a 64–88% and 63–76% reduction in biofilm biomass at 2.3 µg/mL and 1.2 µg/mL, respectively. Furthermore, SeNPs suppressed 95, 97, 99, 94, 99.9, and 99.9% of lasI, lasR, rhlI, rhlR, pqsA, and pqsR expression in *P. aeruginosa* PA01, respectively.

Recent technological advances have strengthened the application potential of Se NPs. For example, Asadpour et al. [206] demonstrated that amikacin-loaded SeNPs exhibited enhanced antibiofilm activity against MRSA isolated from bovine mastitis. Treatment with sub-MIC concentrations of SeNPs inhibited biofilm formation by 55–72%. In preformed biofilms, SeNPs achieved a 2.2–3 log_10_ CFU/mL reduction in viable cells, significantly surpassing the effects observed with free amikacin. Moreover, real-time PCR analysis showed that SeNPs downregulated the expression of key biofilm-associated genes such as intercellular adhesin A (icaA), and intercellular adhesin D (icaD) by more than 50%, thus indicating interference with the genetic regulation of biofilm formation. A study by Annamalai et al. [207] investigated the antibiofilm potential of SeNPs synthesized using the cell-free extract of *Shewanella* sp., a moderately halophilic bacterium isolated from Pichavaram mangrove soil. The synthesized SeNPs significantly reduced *P. aeruginosa* biofilm formation, achieving 85% inhibition at concentrations above 20 μg/mL. This biofilm-disrupting activity was attributed to the low nanoparticle size (~49 nm), which likely facilitated effective penetration through the extracellular matrix and enhanced interactions with bacterial surfaces. The authors also noted that this superior antibiofilm efficiency was influenced by the physicochemical characteristics of the SeNPs and their stable biosynthetic capping. These environment friendly methods yield SeNPs that are naturally coated with bioactive compounds, thereby enhancing both their stability and microbial targeting.

Recently, surface engineered nanoparticles exhibit greater affinity for biofilm embedded bacteria, improving their penetration into the EPS network and amplifying their antibiofilm action. Another promising direction involves the development of SeNPs-doped nanomaterials such as Se/metal–organic frameworks or Se with natural phytochemical-based vehicles such as honey and polyphenols [207,208,209]. These composite systems have shown synergistic effects, resulting in more effective biofilm disruption and stronger suppression of quorum sensing activities. For example, nano selenium-reduced graphene oxide nanocomposite significantly outperformed individual components in preventing biofilm formation by *S. aureus* and *P. aeruginosa* [210]. In another study by Prateeksha [211], honey polyphenol-conjugated SeNPs inhibited quorum sensing in *P. aeruginosa* by downregulating key QS genes like lasR and rhlR, leading to reduced biofilm formation and virulence. Their antioxidant and anti-QS properties enable targeted biofilm suppression without inducing antimicrobial resistance.

In addition to their therapeutic use, SeNPs are being investigated for incorporation into the surface coatings of medical devices such as catheters, orthopedic implants, and wound dressings, to prevent biofilm formation [212]. Such coatings inhibit microbial colonization and biofilm development without adversely affecting surrounding tissues, representing a proactive strategy for infection control. SeNPs at a concentration of 0.5 mg/mL inhibited MRSA adherence by over 60% on both glass and catheter surfaces, highlighting their potential for preventing biofilm formation on medical devices [213].

### 9.4. Antibiofilm Efficacy of Biosynthesized PtNPs

PtNPs represent a promising approach for eradicating MDR biofilms by combining catalytic oxidative damage, biofilm matrix disruption, and quorum sensing inhibition with the added benefit of antibiotic potentiation. These multimodal strategies play a key role in inhibiting MDR biofilms. Acting as nanozymes, PtNPs mimic the catalytic behavior of natural enzymes including peroxidases, catalases, and superoxide dismutase, thereby enhancing their antibacterial and antibiofilm efficacy (Figure 5B) [176,214].

PtNPs exert antibiofilm effects through multiple stages of biofilm development. During initial adhesion, PtNPs alter the physicochemical properties of the surface, reducing microbial attachment through electrostatic interference and localized oxidative stress. In established biofilms, their nanoscale dimensions and potent catalytic activity facilitate their deeper penetration into the EPS, where they disrupt cellular integrity via sustained ROS development [215]. Furthermore, PtNPs modulate intracellular redox balance and disrupt quorum sensing networks, impairing the regulation of biofilm maturation and virulence, and ultimately compromising biofilm resilience at its core. For example, *Desmostachya bipinnata*-mediated green synthesized PtNPs demonstrated 70% inhibition of biofilm formation by *Streptococcus mutants*, *Enterococcus faecalis*, *S. aureus*, and *E. coli*. Further, the synthesized PtNPs generated ROS and downregulated the glucosyltransferase B (GTFB) gene in *S. mutans*, thereby reducing its biofilm ability. PtNPs exhibit potent antibiofilm activity against fungal pathogens through multiple synergistic mechanisms [216]. For example [89], PtNPs synthesized using *X. strumarium* leaf extract exhibited potential inhibition against *C. albicans*, *C. tropicalis*, *C. parapsilosis*, *Aspergillus flavus*, and *Aspergillus niger*. PtNPs directly disrupt the fungal cell membrane particularly targeting ergosterol components, which increases membrane permeability and results in cellular leakage and lysis. Moreover, PtNPs inhibit the initial stages of biofilm formation by interfering with fungal adhesins like agglutinin-like sequence (ALS) family proteins and reducing surface attachment. They further modulate the expression of key biofilm-related genes such as ergosterol 14-alpha demethylase (ERG11), hyphal wall protein 1 (HWP1), ALS3, biofilm and cell wall regulator 1 (BCR1), and enhanced filamentous growth 1 (EFG1), which are involved in adhesion, matrix production, and biofilm maturation. By degrading or inhibiting the synthesis of EPS, including β-glucans, mannans, and chitin, PtNPs destabilize the biofilm matrix and promote its disintegration. In addition, PtNPs may interfere with quorum sensing pathways by disrupting signaling molecules like farnesol, thus hindering fungal communication and coordination during biofilm development. Notably, PtNPs exhibit synergistic effects when combined with conventional antifungal agents by enhancing drug penetration and efficacy.

Advancements in the use of PtNPs for biofilm inhibition have introduced several innovative strategies that enhance their therapeutic potential. Similarly to SeNPs, a prominent approach involves formulating synergistic nanocomposites by combining PtNPs with materials such as graphene oxide (GO). These composites like graphene oxide/platinum (GO/Pt) core–shell nanoparticles demonstrated enhanced antibiofilm activity against *E. faecium* and *K. pneumoniae* [217]. Their proposed mechanism involves the inhibition of EPS, thereby impairing biofilm formation. Additionally, these NPs facilitate the transport of water and nutrients across the polysaccharide layers of the bacterial cell wall. By penetrating these layers, GO/Pt NPs disrupt the structural integrity of the biofilm, ultimately preventing its establishment. Additionally, PtNPs are being integrated into photothermal platforms that exploit hyperthermia-amplified biocatalysis, allowing for biofilm disruption through a combination of thermal and catalytic effects.

To improve the antibiofilm performance of PtNPs, researchers have explored targeted delivery strategies that enhance microbial specificity and reduce off-target effects. One effective approach involves decorating PtNPs with bio-recognition elements such as peptides, antibodies, or small molecules that enable selective interaction with biofilm-associated pathogens. These modifications not only facilitate the better accumulation of PtNPs at infection sites but also increase their retention and penetration within the EPS. This targeted strategy enhances the therapeutic index of PtNPs while minimizing potential cytotoxicity to host cells [218]. Importantly, their mechanism of action based on oxidative stress and structural disruption makes it difficult for pathogens to develop resistance, thus positioning PtNPs as a promising tool in the fight against MDR biofilm-related infections. Table 4 provides a comprehensive overview of biologically synthesized MNPs and their antibiofilm mechanisms, highlighting their potential against pathogens.

## 10. Conclusions

Drug-resistant biofilm infections continue to pose a significant obstacle in clinical treatment due to their high tolerance to conventional antimicrobial agents. In response to this growing concern, MNPs synthesized through eco-friendly and biologically derived processes have garnered considerable interest for their broad-spectrum antibiofilm activity. This review provides a critical synthesis of current progress in the green synthesis, characterization, and therapeutic potential of Ag, Au, Se, and Pt NPs. Bioinspired fabrication methods, particularly those employing plants, microbes, and biomolecules offer a sustainable and biocompatible route for nanoparticle production, minimizing environmental and toxicological concerns associated with traditional methods.

Each type of MNPs displays distinct mechanisms of antibiofilm action: AgNPs generate oxidative stress and interfere with quorum sensing; AuNPs serve as carriers for targeted drug delivery and photothermal disruption; SeNPs modulate redox balance with minimal cytotoxicity; and PtNPs function as nanozymes with continuous catalytic activity, which are particularly effective against both bacterial and fungal biofilms. These nanoparticles not only inhibit microbial attachment and colonization but also penetrate mature biofilms, destabilize EPS, and sensitize pathogens to antibiotics. Their surface tunability, small size, and ability to interact with microbial cells at multiple levels make them ideal candidates for integrated antimicrobial platforms.

## 11. Future Perspectives

Bioinspired MNPs have emerged as promising candidates in the fight against drug-resistant biofilm-associated infections. Despite considerable advancements in their synthesis and preliminary applications, their translation from laboratory studies to clinical practice remains challenging. To move forward, future research must focus on refining production methods, uncovering precise bio-interactions, enhancing their functionality, validating their safety, and navigating regulatory frameworks.

### 11.1. Toward Scalable and Consistent Green Synthesis

One of the main hurdles in the development of bio-fabricated MNPs is the variability associated with biological raw materials. Differences in phytochemical profiles due to plant species, growth conditions, and extraction techniques can lead to inconsistencies in nanoparticle characteristics such as morphology, size distribution, and stability. Therefore, establishing consistent, and scalable synthesis methods is a priority. The adoption of automated synthesis systems, combined with real-time monitoring tools may support quality assurance. Moreover, computational approaches including artificial intelligence and predictive modeling can aid in optimizing synthesis conditions based on specific biological templates.

### 11.2. Understanding Mechanisms and Improving Target

Although the antibiofilm capabilities of MNPs are well-documented, the underlying molecular interactions are still not fully elucidated. Future studies should integrate omics-based techniques such as genomics, transcriptomics, and metabolomics to identify key microbial pathways targeted by MNPs. Enhanced imaging tools and single-cell analyses can provide further insight into the behavior of nanoparticles within structured biofilms. Additionally, the surface engineering of MNPs with specific ligands, antibodies, or peptides could improve selective targeting, minimizing off-target effects and enhancing therapeutic efficiency.

### 11.3. Development of Multi-Functional Nanoplatforms

The design of advanced nanoplatforms that combine therapeutic and diagnostic functions is gaining momentum. Such systems could enable the real-time detection of biofilms along with localized treatment. Smart nanocarriers responsive to specific stimuli such as changes in pH, enzyme activity, or redox conditions can be employed for site-specific drug release. Hybrid systems that incorporate MNPs into polymeric, liposomal, or carbon-based matrices may further improve penetration into dense biofilm matrices and allow for controlled and sustained action against embedded pathogens.

### 11.4. In Vivo Models and Safety Profiling

While most current data are derived from in vitro studies, in vivo validation remains essential to assess the safety and therapeutic performance of MNPs under physiologically relevant conditions. Animal models that replicate chronic and device-associated infections such as murine wound biofilm models and catheterized implants should be utilized. Furthermore, comprehensive safety studies evaluating nanoparticle clearance, accumulation, immune response, and long-term effects are critical. Early-stage toxicity screenings can benefit from the use of model organisms like zebrafish embryos and insect larvae to supplement traditional mammalian systems.

### 11.5. Navigating Regulatory and Commercialization Challenges

The path to clinical translation involves more than scientific validation; it requires regulatory clarity and industry collaboration. The current regulatory guidelines for nanoparticle-based products are still evolving. As such, researchers should work closely with regulatory bodies to establish robust safety, efficacy, and quality control standards. Parallel efforts should focus on developing cost-effective and Good Manufacturing Practice (GMP)-compliant production methods to facilitate industrial-scale manufacturing. Building interdisciplinary partnerships will be vital to support technology transfer and accelerate commercial readiness. The future of MNP-based antibiofilm strategies lies in integrating sustainable production, targeted design, functional innovation, and rigorous safety evaluations. By addressing current limitations through cross-disciplinary research and regulatory engagement, bioinspired MNPs hold the potential to redefine the treatment paradigm for persistent biofilm infections.

## Figures and Tables

**Table 1:** Plant-mediated synthesis of MNPs: type, shape, and size characteristics.

Plant Name	Plant Part Used	Type of NPs	Shape	Size (nm)	References
*Antigonon leptopus*	Leaf	Ag	Spherical	4–20	Gastelum-Cabrera et al. [64]
*Harrisonia abyssinica*	Fruit	Ag	Spherical	2–24	Mwakalesi et al. [65]
*Tinospora cordifolia*	Leaf	Ag	Spherical	125	Lekkala et al. [66]
*Punica granatum*	Seeds	Ag	Spherical	10–35	Muthu et al. [67]
*Melia azedarach*	Leaf	Ag	Spherical	18–30	Jebril et al. [68]
*Eichhornia crassipes*	Leaf	Ag	Spherical, cubic, rod and hexagonal	57.93, 56.44 and 58.25	Heikal et al. [69]
*Moringa oleifera*	Flower	Ag	Spherical	8	Bindhu et al. [70]
*Clitoria ternatea*	Flower	Ag	Quasi-spherical	3.21–43.7	Singh et al. [71]
*Calotropis procera*	Root	Ag	Spherical and square	38–44	Sagadevan et al. [72]
*Cyperus conglomeratus*	Root	Ag	Spherical	70–100	Al-Nuairi et al. [73]
*Tamarindus indica*	Fruit	Ag	Spherical	20–52	Gomathi et al. [74]
*Vitis vinifera*	Fruit waste	Ag	Spherical	22–35	Saratale et al. [75]
*Brassica oleracea* var. *botrytis*	Flower waste	Ag	Spherical	5–50	Kadam et al. [76]
*Apium graveolens*	Leaf and stem	Au	Spherical	4–15	Panchamoorthy et al. [77]
*Jasminum auriculatum*	Leaf	Au	Spherical	8–37	Balasubramanian et al. [78]
*Cucurbita moschata*	Fruit peel	Au	Spherical	18.10	Kaval and Hosgoren [79]
*Hibiscus sabdariffa*	Flower	Au	Spherical	15–45	Zangeneh et al. [80]
*Lonicera japonica*	Flower	Au	Spherical, triangular and hexagonal	10–40	Patil et al. [81]
*Lycium chinense*	Fruit	Ag and Au	Spherical	50–200 and 20–100	Chokkalingam et al. [82]
*Euphorbia fischeriana*	Root	Au	Core–shell	20–60	Zhang et al. [83]
*Codonopsis pilosula*	Root	Au	Spherical	20 ± 3.2	Doan et al. [84]
*Tinospora cordifolia*	Stem	Au	Spherical	16.1	Ali et al. [85]
*Azadirachta indica*	Leaf	Se	Spherical	142–168	Mulla et al. [86]
*Dillenia indica*	Leaf	Se	Oval	50–900	Krishnan et al. [87]
*Phoenix dactylifera*	Fruit	Pt	Quasi- spherical	2.3–3	Al-Radadi et al. [88]
*Xanthium strumarium*	Leaf	Pt	Cubic	22	Kumar et al. [89]
*Combretum erythrophyllum*	Leaf	Pt	Spherical	1.04 ± 0.26	Fanoro et al. [90]
*Nigella sativa*	Leaf	Pt	Spherical	3.47 ± 1.31	Aygun et al. [91]

**Table 3:** Biomolecule-mediated synthesis of MNPs: type, shape, and size characteristics.

Biomolecules	Type of NP	Shape	Size (nm)	References
Chitin	Ag	Rod	15–70 length and 5–10 breadth	Vijayaraj et al. [137]
Curcumin-chitosan	Au	Spherical	128.27	Zainol Abidin et al. [138]
Carrageenan/nanocellulose	Ag	Spherical	20–200	Jaffar et al. [139]
*Fructus aurantii*-loaded Citrus pectin	Ag	Spherical	5–32	Chang et al. [140]
Chitosan	Au	Spherical	45–60	Alsadooni et al. [141]
Pectin	Au	Spherical	14	Borker and Pokharkar [142]
Rutin/chitosan	Ag	Spherical	23–78	Bharathi et al. [143]
Pectin	Se	Spherical	~61	Wu et al. [144]
Gum kondagogu	Se	spheroids	44.4–200	Kora [145]
L-cystine	Se	Spherical	60	Prasanth and Sudarsanakumar [146]
*Prunus × yedoensis*—gum	Pt	Circular	10–20	Velmurugan et al. [147]
Gum arabic	Pt	Spherical	6–10	Elamin et al. [148]
Lichenan from *Usnea longissima*	Se	Spherical	76	Yang et al. [149]

**Table 4:** Summary of biologically synthesized MNPs as well as their targeted biofilm-forming pathogens, proposed antibiofilm mechanisms, and relevant literature references.

Nanoparticles	Synthesis Source	Antibiofilm Activity	Proposed Mechanism	References
Ag	*Deinococcus radiodurans*	*E. coli* and *S. aureus*	Generation of ROS, leading to oxidative stress that damages bacterial DNA and proteins, resulting in protein denaturation and disruption of metabolic processes	Velmathi et al. [188]
Au	Baicalein	*P. aeruginosa* PAO1	Inhibits EPS synthesis, reducing the structural integrity of biofilms and impairing bacterial adhesion and communication.	Rajkumari et al. [219]
Ag	*Oscillatoria* sp.	*E. coli* 35218, *S. aureus*, *P. aeruginosa*, *Citrobacter*, *S. typhi*, *E. coli* 11775, and *Bacillus* sp.	Interferes with bacterial replication by deactivating DNA-binding enzymes, thus preventing DNA synthesis and cell division.	Adebayo-Tayo et al. [220]
Ag	*Bacillus licheniformis* Dahb1	*V. parahaemolyticus* DAV1	Triggers premature detachment of bacterial cells by disrupting biofilm matrix stability, likely through oxidative stress and signaling interference.	Shanthi et al. [221]
Ag/Au bimetallic NPs	*Gloriosa superba*	*S. aureus*, *S. pneumoniae*, *K. pneumoniae*, and *E. coli*	Disrupts bacterial cell membrane integrity, causing leakage of intracellular contents and loss of membrane potential essential for cell viability.	Gopinath et al. [222]
Ag-CS nanocomposite	*Streptomyces* strain RB7AG and chitosan	*E. coli* and *S. aureus*	Induces ROS production and disrupts membrane permeability, enhancing chitosan’s cationic interaction with bacterial surfaces for effective biofilm penetration.	Behera et al. [223]
Au	Jellein-I peptide conjugated	MRSA	Interferes with protein function by binding to key enzymes and structural proteins in MRSA, leading to impaired biofilm architecture and cellular metabolism.	Sattari-Maraji et al. [224]
Au	Cinnamaldehyde	*C. albicans*	Disrupts biofilm formation by inhibiting hyphal growth and morphogenesis in *C. albicans.*	Ramasamy et al. [193]
Au	*Artocarpus heterophyllus*	*A. baumannii* and MRSA	Inhibits initial biofilm establishment by blocking bacterial adhesion and proliferation, likely by targeting surface adhesins and cell division proteins.	Hudaya et al. [225]
Ag-Se	*Orobanche aegyptiaca*	*S. aureus*	Suppresses the production of exopolysaccharides by interfering with bacterial signaling pathways that regulate EPS biosynthesis.	Mostafa et al. [226]
Au	*Capsicum annum*	*P. aeruginosa* PAO1	Inhibits quorum-sensing networks responsible for the regulation of virulence genes and biofilm maturation in *P. aeruginosa*.	Qais et al. [195]
Se	*Providencia vermicola* BGRW	*S. aureus*, *B. cereus*, *E. coli*, *Proteus* sp. *P. aeruginosa*, and *S. enteritidis*	Disrupts the structural matrix of mature biofilms by degrading the glycocalyx and inhibiting biofilm matrix cohesion.	El-Deeb et al. [227]
Se	*B. subtilis* BSN313	*P. aeruginosa* ATCC 9027, *S. typhi* ATCC 14028, and *S. aureus* ATCC 25923	Alters bacterial surface properties such as hydrophobicity and electrostatic interactions, hindering cell aggregation and biofilm integrity.	Ullah et al. [228]
Se	*Pseudomonas aeruginosa* OG1	*S. salivarius* and *P. mirabilis*	Reduces biofilm formation by inhibiting oxidative stress response, impairing bacterial adhesion and biofilm stabilization.	Gurkok et al. [229]
Au/Pt/Ag	Lamii albi flos	Enterococcal strain	Targets sessile bacterial populations by penetrating biofilms and disrupting intracellular processes, leading to the eradication of planktonic escape cells.	Dlugaszewska et al. [230]
Pt	*Desmostachya bipinnata*	*S. aureus*, *Enterococcus faecalis*, and *S. mutants*	Promotes intracellular ROS accumulation, resulting in oxidative damage to essential biomolecules and impairment of metabolic pathways in multidrug-resistant pathogens.	Krishnasamy et al. [216]

## Data Availability

Data are contained within the article.
